# Molecular Link between Vitamin D and Cancer Prevention

**DOI:** 10.3390/nu5103993

**Published:** 2013-09-30

**Authors:** Meis Moukayed, William B. Grant

**Affiliations:** 1 School of Arts and Sciences, American University in Dubai, P. O. Box 28282, Dubai, UAE; E-Mail: mmoukayed@hotmail.com; 2 Sunlight, Nutrition, and Health Research Center, San Francisco, CA 94164-1603, USA

**Keywords:** vitamin D, cancer, mechanisms, ecological studies, observational studies, prevention, animal models

## Abstract

The metabolite of vitamin D, 1α,25-dihydroxyvitamin D_3_ (also known as calcitriol), is a biologically active molecule required to maintain the physiological functions of several target tissues in the human body from conception to adulthood. Its molecular mode of action ranges from immediate nongenomic responses to longer term mechanisms that exert persistent genomic effects. The genomic mechanisms of vitamin D action rely on cross talk between 1α,25-dihydroxyvitamin D_3_ signaling pathways and that of other growth factors or hormones that collectively regulate cell proliferation, differentiation and cell survival. *In vitro* and *in vivo* studies demonstrate a role for vitamin D (calcitriol) in modulating cellular growth and development. Vitamin D (calcitriol) acts as an antiproliferative agent in many tissues and significantly slows malignant cellular growth. Moreover, epidemiological studies have suggested that ultraviolet-B exposure can help reduce cancer risk and prevalence, indicating a potential role for vitamin D as a feasible agent to prevent cancer incidence and recurrence. With the preventive potential of this biologically active agent, we suggest that countries where cancer is on the rise—yet where sunlight and, hence, vitamin D may be easily acquired—adopt awareness, education and implementation strategies to increase supplementation with vitamin D in all age groups as a preventive measure to reduce cancer risk and prevalence.

## 1. Vitamin D: Introduction, Function and Metabolism

### 1.1. Introduction to Vitamin D: History and Physiological Roles

Vitamin D refers to two fat soluble substances, vitamin D_3_ (cholecalciferol) and vitamin D_2_ (ergocalciferol), and their metabolites, which are considered to be important nutrients for human health. Dietary vitamin D_3_ sources include dairy, eggs, fish and meat [1], while dietary vitamin D_2_ (ergocalciferol) sources are UVB-irradiated yeast and fungi [2,3]. However, there seem to be some unaccounted for dietary sources [4]. In humans, vitamin D_3_, made naturally by the body following exposure to ultraviolet light, acts as an important endocrine hormone precursor.

**Figure 1:**
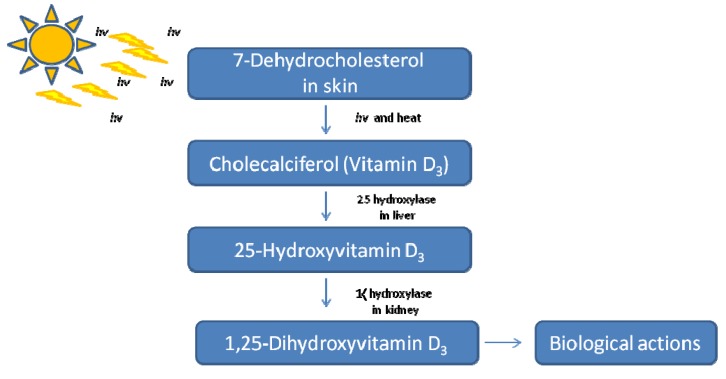
Ultraviolet radiation (290–330 nm) from the sun and body heat convert 7-dehydrocholesterol in the skin to cholecalciferol (vitamin D_3_). Vitamin D_3_ is further metabolized in the liver and kidneys to yield the active metabolite 1,25(OH)_2_D_3_.

Mellanby [5] and McCollum [6] first identified vitamin D’s effects in experiments that investigated the chemical components of cod liver oil that could prevent rickets in animals. Later studies by Hess [7], McCollum [8], Steenbock [9], Askew [9] and Windaus [10] helped isolate, identify and determine the structure and function of this hormone and its essential role in skeletal health. In 1939, Windhaus determined the structure and initial pathways by which vitamin D_3_ was synthesized from 7-dehydrocholesterol [11,12]. His biochemical investigations were the basis for Holick and colleagues [13,14,15,16] to later elucidate the biochemical pathways and physiological mechanisms that regulated the formation of the active vitamin D hormone, 1α,25-dihydroxyvitamin D_3_ [17]. Vitamin D_3_ is initially synthesized via the initial conversation of 7-dehydrocholesterol upon UV irradiation of the skin [13,14,15,16]. This provitamin D_3_ is further metabolized in the liver and kidneys to produce the active hormone, 1α,25-dihydroxyvitamin D_3_, often mentioned in the literature simply as vitamin D [18,19,20,21] (Figure 1). However, extrarenal production of 1,25-dihydroxyvitamin D also occurs in many organs [22]. 25-hydroxyvitamin D [25(OH)D] is the circulating metabolite of vitamin D that is routinely measured.

In the 90 years since the above discovery to date, scientists have demonstrated unequivocally that vitamin D exerts a spectrum of biological effects well beyond its classical role in calcium and phosphate homeostasis. The cloning of the vitamin D receptor (VDR) in 1987 and the detection of VDRs in almost all tissues of the body spurred wide interest in its physiological functions [23]. Vitamin D_3_ has important homeostatic functions in fetal and adult development and differentiation in endocrine, metabolic, neurological, epidermal and immunological systems of the human body [24,25,26] (Table 1). Moreover, several studies support an essential role for vitamin D in regulating mechanisms controlling cell proliferation, differentiation and growth. These bodies of evidence reveal protective functions for vitamin D against carcinogen-induced neoplasia and recurrent secondary metastasis [27].

**Table 1:** Vitamin D can exert its action in several organ systems and tissues of the body. This occurs in a paracrine, autocrine, intracrine or endocrine manner [24,25,26].

Organ or system	Target tissue or cell	Specific effects
Intestine	Duodenum	↑ Intestinal calcium absorption (TRPV6 intestinal calcium transporters) ↑ Calbindin D28k
	Jejunum (brush border and basolateral membranes)	↑ Intestinal phosphate transport
Bone	Osteoblasts (and, in turn, osteoclasts) and chondrocytes	↑ Bone formation: bone mineralization and matrix formation; ↑ osteocalcin; ↑ osteopontin/SPP1; ↑ RANKL for osteoblasts to activate osteoclasts
Parathyroid gland	Chief cells	↓ PTH
Kidneys	Distal tubules (Ca) Proximal Tubules (phosphate)	↑ Reabsorption of Calcium (↑ TRPV5, calbindin) ↑ Reabsorption of phosphate (↑ NPT1 and NPT2) ↑ Detoxification of 1α,25 dihydroxyvitamin D_3_ (CYP24A1 OHase) ↑ Calbindin D9k
Immune system	Monocytes/macrophages and T lymphocytes (helper type 1)	Suppression of γ-interferon and IL-1–6
Central nervous system	Dorsal root ganglia (glial cells) and hippocampus	↑ Production of NGF, neurotrophin 3 and leukemia-inhibitory factor
Epithelium	Epidermal skin (keratinocyte)	↑ Differentiation
	Hair follicle	↑ Differentiation
	Female reproductive tract	Uterine development
	Mammary	↓ Cell growth
	Prostate	↓ Cell growth
	Colon	↓ Cell growth
Endocrine target tissues	Thyroid gland	↓ TSH
	Pancreatic β-cells	↑ Insulin secretion (Calbindin 28K)
Many systems	Diverse cells and cancer cell lines	↓ Cell growth (↓ c-Fos, ↓ c-Myc) ↑ Differentiation (↑ p21, ↑ p27) ↑ Apoptosis (↓ *Bcl-2*)↓ Angiogenesis

↑ = upregulates/increases; ↓ = downregulates/decreases. CYP24A1 OHase = 1,25-dihydroxyvitamin D_3_ 24-hydroxylase; IL = interleukin; NGF = nerve growth factor; NPT1 = sodium-dependent phosphate transporter 1; NPT2 = sodium-dependent phosphate transporter 2; PTH = parathyroid hormone; RANKL = receptor activator of nuclear factor kappa-B ligand; SPP-1 = secreted phosphoprotein 1; TRPV5 = transient receptor potential cation channel subfamily V member 5; TRPV6 = transient receptor potential cation channel subfamily V member 6; TSH = thyroid stimulating hormone.

### 1.2. Vitamin D Genomic Action via the VDR

#### 1.2.1. VDR Distribution, Dimerization and Function

Vitamin D acts as a steroid hormone by regulating gene transcription. The effect of the bioactive hormone 1α,25-dihydroxyvitamin D_3_ occurs via the VDR. The VDR was identified in 1969 [28] and was cloned in 1987 [29]; its crystal structure and binding to its natural ligand were characterized in 2000 [30]. Since its discovery, researchers have detected the VDR in many tissues of the body, including bone, pancreatic β cells, parathyroid gland, brain, skin, prostate, testes, heart, skeletal muscle tissue, breast, liver, lung, intestine, kidneys, adipose cells and immune response cells, such as macrophages, dendritic cells and activated B- and T-cells [31,32,33,34,35,36]. The receptor is widely distributed in the body, indicating physiological roles in homeostatic regulation beyond bone tissue mineralization. Ligand binding by 1α,25-dihydroxyvitamin D_3_ to its receptor can either activate or repress genes [37,38,39,40,41]. The VDR exerts its genomic effects by binding to regulatory vitamin D response elements (VDREs) present in the promoter regions of target genes in these tissues. The most prevalent motif for the VDRE sequence consists of two half-sites, each with the six-nucleotide consensus sequence, GGTCCA, separated by three other nucleotides of any sequence [37]. This motif is known as direct repeat 3 (DR3), although other configurations of VDRE-binding sites, including DR6 and DR4, exist in some vitamin D-regulated genes [26,37,42,43].

Ligand-bound VDR-dependent transcription regulation of genes occurs via the dimerization of the receptor with the retinoic X receptor (RXR). Although VDR preferentially binds to RXR, creating a VDR-RXR dimer, VDR can also bind other receptors of the nuclear receptor superfamily, which include thyroid, vitamin A, PPAR-γ and other orphan receptors [44]. The VDR-RXR dimer can regulate genes in several systems and tissues [26].

#### 1.2.2. VDR Structure and Role of Cofactors

The VDR has two variant isoforms, one with 424 amino acids (aa) and the other with 427 aa [45,46]. The complete functional protein contains several distinct domains: a DNA-binding domain (aa 24–115), a nuclear localization sequence (aa 44–55, 79–105), a hinge domain (aa 116–226), a dimerization domain (aa 37, 91–92, 244–263, 317–395) that overlaps with the ligand-binding domain (LBD) (aa 227–244, 268–326, 396–422) and the sequences of the activation domain (AF2; aa 246, 416–422) [47,48].

As with other nuclear hormone receptors, VDR has a conserved DNA-binding domain that comprises two zinc fingers necessary for target gene VDRE binding and dimerization of the activated receptor. The first zinc finger (proximal to the N terminus of the protein) is required for docking at the VDRE, whereas the second zinc finger is required for binding dimerization with RXR. A nuclear localization sequence is located within the DNA-binding region and just upstream of the *C* terminal to this binding domain. The nuclear localization sequence directs localization of the activated receptor to the nucleus to regulate gene transcription. The LBD, which consists of 12 α-helices joined by β-sheets, has regions required for 1α,25-dihydroxyvitamin D_3_ binding at helix 2 and two further regions required for RXR heterodimerization (see [30,37] for key references on LBD crystal structure and properties and functions). Within this *C*-terminus region of the LBD lies the major activation domain, AF2, required for transcription coactivation. Ligand binding by the active hormone creates a conformational change that releases transcription repressors such as WSTF and WINAC and enables attachment of coactivators [49,50,51]. AF2 is bound by cofactors of the steroid receptor coactivator (SRC) family, NCoA62-SKIP, CBP/p300 and p/CAF, and proteins of the VDR-interacting protein complex (DRIP/Mediator) [52,53]. CBP/p300 and p/CAF have histone acetyl transferase ability, which unravels the chromatin of the target gene [54]. This unwinding of the chromatin enables the transcription machinery, such as TF2B and RNA polymerase II, to bind and subsequently initiate transcription [55]. The DRIP complex, made up of approximately 15 leucine-rich proteins, is unique in its function in that it creates a bridge between the SRC coactivator proteins lodged at the VDRE of the gene and the transcription machinery at the start site of the regulated gene. DRIP 205 specifically is required in this vitamin D-induced transcription activation to enable an open position of the transcribed chromatin [56,57].

#### 1.2.3. Nongenomic Cytoplasmic Action

Vitamin D can also function via nongenomic mechanisms without exerting transcriptional effects or requiring additional protein synthesis. Such nongenomic effects have previously been reported for other nuclear hormones, such as estrogen, thyroid hormone and corticosteroids [58,59]. Vitamin D is thought to regulate rapid cellular calcium flux and calcium-activated chloride channel activity via a membrane-bound receptor protein known as 1,25-(OH)_2_D membrane–associated rapid response steroid-binding protein (1,25 D-MARRSBP), also known as endoplasmic reticulum stress protein 57. MARRSBP was identified, purified and cloned from chick intestinal epithelium [60]. MARRSBP has also been found in tissues, including osteoblasts, liver, adipocytes and muscle [61,62,63,64,65,66,67]. MARRSBP acts via a G protein-coupled process that activates phospholipase C. Phospholipase C hydrolyzes membrane-bound phosphoinositol bisphosphate (PIP_2_) to release inositol trisphosphate (IP_3_) and diacylglycerol. In turn, these cellular messengers enable the release of calcium from endoplasmic stores and mediate the acute quick release of calcium in the cell [68]. This interaction, however, cannot occur without nuclear-targeted VDR, indicating that a cooperative mechanism of action between membrane-bound MARRSBP and VDR mediates rapid responses in some tissue types [35,69]. This interaction between the membrane-bound and nucleus-bound receptors can occur in cancer cell lines. In MCF-7 breast cancer cells, MARRSBP antagonizes VDR, thus implicating the membrane-bound MARRSBP in the modulation of cellular proliferation and malignancy. However, the full purpose of this interaction is yet to be elucidated [70].

## 2. Vitamin D and Cancer

### 2.1. Vitamin D Mechanisms Regulating Cellular Proliferation and Growth

Through the genomic actions of vitamin D_3_ via VDR regulation of several genes containing VDREs, 1α,25(OH)_2_ vitamin D_3_ and its analogues inhibit cell cycle progression and tumor cell growth in several cancer cell lines. Such mechanisms range from preventing cell proliferation (cell cycle arrest) to inducing apoptosis to inducing or suppressing cell adhesion molecules and growth factors that promote cellular homing and metastasis. 

### 2.2. Cell Cycle and Apoptosis

The cell cycle is regulated by a complex network of interlinked regulators that in concert govern cellular proliferation. Vitamin D may exert growth inhibitory effects through repression of different key molecules involved in cell cycle regulation. For example, Jensen and colleagues [71] demonstrated that 1,a,25-(OH)_2_D_3_ treatment of human breast cancer cell line MCF-7 resulted in repression of c-Myc, a known proto-oncogene in the cell cycle regulatory machinery. Salehi-Tabar and colleagues [72] demonstrated that 1,a,25-(OH)_2_D_3_ can suppress expression of the oncogene c-Myc and, thus, promotes the increased expression of its antagonist, the transcriptional repressor, MAD1/MXD1. This effect is mediated by other cellular regulators, such as F-box protein (FBW7). Meyer and colleagues [73] also demonstrated that in colonic cells of colorectal tumors, treatment with 1,a,25-(OH)_2_D_3_ can suppress c-Fos and c-Myc gene expression in a manner that involves B-catenin signaling interactions. In prostate cancer cells, Washington and colleagues [74] showed that this repression is independent of retinoblastoma protein (Rb). Li and colleagues [75] showed that treatment of ovarian cancer cells with 1,25-dihydroxyvitamin D_3_ results in p27(Kip1) stabilization and G(1) arrest through downregulation of cyclin E/cyclin-dependent kinase 2 and Skp1-Cullin-F-box protein/Skp2 ubiquitin ligase. Collectively, these studies confirm that vitamin D can suppress cell proliferation through inhibitory effects on several regulators in the network of cell cycle control machinery.

Akutsu and colleagues [76] reported that treating human head and neck squamous cell carcinoma cells SCC25 with vitamin D arrested cell proliferation at the G_0_/G_1_ phase. Vitamin D also upregulated the growth-arrest DNA damage repair factor, GADD45α, at both the mRNA and protein levels. Cyclin-dependent kinase inhibitor p21 was also induced at the mRNA level, but not at the protein level. Chiang and colleagues showed that both vitamin D and its analogue, MART-10, were potent chemicals that could arrest the cell cycle at the G_0_/G_1_ transition phase in MCF-1 breast cancer cell lines. This process occurred through the expression of BAX/BCL proapoptotic proteins and the initiation of apoptosis via release of cytochrome C from mitochondria. Moreover, MART-10 induced no observed hypercalcemic effects (which are sometimes observed with vitamin D treatment), suggesting that such analogues would be good agents to deter cancer progression without detrimental side effects ([77]). In adenoma and carcinoma colorectal cell lines (SW620, PC/JW and HT29), treatment with vitamin D or its analogue, EB1089, induced apoptosis independent of p53, increased the number of cells in the G_1_ phase and increased levels of the proapoptotic protein, Bak (a member of the *BCl-2* gene family), in all cell lines tested. This finding suggested that vitamin D and its analogues are clinically effective for treating colorectal cancers. In rat glioma C6.9 cell lines, vitamin D treatment induced apoptosis via DNA fragmentation and upregulation of *p53* and *GADD45* genes [78]. In prostate cancer cell lines, vitamin D treatment inhibited cyclin-dependent kinase 2 activity and induced G_0_/G_1_ cell cycle arrest [79]. Therefore, several lines of evidence from *in vitro* studies support the role of vitamin D in promoting cell cycle arrest and promoting apoptosis in malignant transformed cells.

### 2.3. Hypoxia, Oxidative Stress, HIF-1 and Angiogenesis

Hypoxia and oxidative stress are often associated with cancer progression. Oxidative stress often induces DNA damage and loss of DNA-repair ability [80,81]. Hypoxia also promotes hypoxia-inducible factor 1 (HIF-1)-dependent angiogenesis essential for tumor growth [82]. Treatment of several cell lines, such as SW-480-ADH, LNCaP (a prostate cancer cell line) and MCF-7, with vitamin D activates cellular signaling cascades that reduce thioredoxin and promote antioxidant responses, induce mRNA expression of superoxide dismutase (in prostate epithelial cells) and downregulate glutathione levels by increasing glucose-6-phosphate dehydrogenase expression [83,84,85].

Vitamin D_3_ inhibits initiators of cellular angiogenesis in several cancer cell lines. Adding vitamin D_3_ to the highly aggressive androgen-insensitive prostate cancer cell line, CL-1, inhibits proliferation of these cells both in normoxia and in hypoxic environments that resemble those in cancer tissues. This effect is also observed in LNCaP and in SW-480 colon cancer cell lines. Vitamin D_3_ also inhibits secretion of vascular endothelial growth factor (VEGF) in these cell lines, as well as in the MCF-7 breast cancer cell line. Furthermore, vitamin D treatment downregulates endothelin 1 (ET-1) and glucose transporter 1 (Glut-1). VEGF-1, ET-1 and Glut-1 are essential for inducing angiogenesis. This molecular effect is mediated via significant downregulation of HIF-1 transcription and translation [86].

A study by Chung and colleagues [87] showed that in tumor-derived endothelial cells from VDR knockout mice, loss of VDR resulted in an increase in HIF-1α, VEGF, angiopoietin 1 and platelet-derived growth factor levels. Moreover, *in vivo*, mice lacking VDR exhibited enlarged blood vessels to perfuse tumor lesions. This clearly implicates VDR in the control of tumor-associated angiogenesis.

### 2.4. Interactions with Growth Factors that Mediate Transformation, Cell Adhesion and Metastasis

Vitamin D_3_ strongly inhibits the Sonic Hedgehog signaling cascade in human renal cell carcinoma. Mice xenografted with renal cell carcinoma cells yet treated with vitamin D exhibit the absence of tumor development or substantial growth inhibition, suggesting the vitamin D_3_ can be used prophylactically to prevent tumor development or regression [88]. 

Insulin-like growth factors (IGFs) and their binding proteins have been implicated in the development of several tumors [89]. In prostate cancer, for example, IGFBP3 protein levels decrease when cells progress from benign to malignant metastasis [90,91]. Microarray analysis has shown that vitamin D_3_ regulates IGFBP3, which sequesters and modulates levels of IGF-I, in LNCaP human prostate cancer cell lines [92]. Upon treatment with vitamin D_3_, IGFBP3 mRNA is highly upregulated in LNCaP cells. In malignant and metastatic MCF10CA breast cancer cells, IGFBP3 transcripts were upregulated after treatment with a vitamin D analogue, Gemini [93]. IGFBP5 gene expression was induced after treatment with vitamin D_3_ in MCF-7 breast cancer cell lines [94].

An interaction in cellular signaling that is required for vitamin D’s growth-inhibitory role in prostate cancer cells occurs between vitamin D and androgens [79]. Without androgen receptors, vitamin D is ineffective in its protective action, indicating that the protective function of the hormone requires signaling cross talk between both molecules.

Similarly, in the colon cancer cell line, Caco-2, vitamin D signaling is thought to modulate the apoptotic effects of transforming growth factor β1 (TGF-β1). Unlike its effect on normal epithelial cells, TGFβ cannot inhibit cell growth in Caco-2 (and other colon cancer-derived cell lines, such as SW-480). However, this resistance is reverted upon treatment of Caco-2 cell lines with vitamin D [95]. The effect of vitamin D_3_ appears to be via upregulation of IGF-II receptors and increased expression of TFG-β1 itself in these cell lines. Furthermore, the apparent sensitization of cells to apoptosis by vitamin D in otherwise apoptosis-resistant malignant cell lines indicates an important role for vitamin D in the treatment of resistant refractory tumors. Vitamin D interacts with TGF-β–SMAD1 signaling, blocks transcriptional expression of cell cycle proteins and inhibits the action of cell cycle protein cyclins D_1_, D_2_, D_3_ and E. Vitamin D can also inhibit epidermal growth factor signaling and its mitogenic Ras signaling [40,96,97].

Wnt signaling activation is a key culprit in the pathogenesis of several cancers [98,99,100,101]. Wnt acts through the nuclear localization of β-catenin and its association with downstream transcription factors, such as TCF1 (also known as TCF7) and TCF4 (also known as TCF7L2). Upon nuclear localization, β-catenin can activate several genes involved in tumor growth, metastasis or angiogenesis. Wnt can upregulate cell cycle progression genes, such as cyclin D, c-Myc and c-jun, matrix metalloproteinase MMP-7 [102], and limb, bud and heart (LBH) transcription factor, which promote cell malignancy and metastasis. Wnt also upregulates endothelin 1, VEGF and interleukin 8 (IL-8), all of which promote angiogenesis, required to feed aggressive tumors [103,104,105]. Wnt also downregulates E-cadherin, required for cellular adhesion [106]. This downregulation may modulate the metastatic transformation associated with Wnt-activated tumor cells [107]. The antitumor effects of vitamin D that antagonize Wnt signaling action have been best demonstrated in breast, colorectal and prostate cancer cells. Vitamin D treatment downregulates several of the above tumorigenesis-promoting genes. This action is modulated by the genomic effects of VDR interaction with β-catenin and, therefore, suppression of Wnt-activated transcription induction of the above-mentioned genes [85,94,107,108,109,110,111,112]. Vitamin D also upregulates the Wnt antagonist, DKK-1, thus suppressing Wnt activation and associated transformation [112,113].

Vitamin D treatment blocks production of IL-1β in macrophages [114] and, hence, blocks inflammation associated with colon carcinoma progression. This inactivation of IL-1β suppresses inflammation and, in turn, Wnt signaling activation in colon cancer epithelial cells required for the progression of colon tumors [115]. The suppressive effects of vitamin D on inflammation have also been confirmed through studies that have shown that vitamin D can suppress IL-1β, IL-6 and IL-17 and NF-κB in inflammation associated with breast and prostate cancer cells [107,116,117].

### 2.5. Autophagy

Vitamin D regulates cancer-associated autophagy. Autophagy is important in the prevention of tumor progression *in vivo* [118,119]. Autophagy allows cells to survive in conditions of stress, such as low oxygen or nutrient deprivation, through digestion of cellular debris or accumulated damaged organelles. Such debris may damage the cell and, hence, affect viability and survival. Although it may be considered a contradiction to accept that promoting cell survival in tumor cells would be warranted, studies in HL60 myeloid leukemia cells and in MCF-7 breast cancer cell lines indicate that activating autophagy pathways by the hormone, vitamin D, is important in exciting signaling pathways mediating the tumor-suppressive role of vitamin D. Vitamin D treatment of tumor cells seems to mediate upregulation of a protein known as beclin-1, which interacts with PI3 kinase (PI3K), which, in turn, inhibits mTOR, responsible for promoting tumor growth and progression. The specific inhibition of mTOR signaling is one that suppresses molecules, such as CDK inhibitors, p19 and p27, but is independent of p53-mediated mechanisms. Vitamin D treatment is crucial for maintaining cell viability and survival via signaling pathways that require PI3K and mitogen-activated protein kinase signaling and that are independent of pathways eliciting apoptosis. Such signaling activation is effective at early stages of cellular damage, which is associated with carcinogenesis (and cancer therapy), and where neovascularization of tissues is absent. Treatment with vitamin D in such tumors at early stages of cellular transformation would induce autophagy with the aim of clearing damaged chromosomes and organelle debris [120]. After autophagy, p53 and other tumor-suppressor genes can either initiate cellular repair cascades if tumorigenesis is in its earlier stages or induce apoptosis in cells sensitized for treatment with radiotherapy or chemotherapy [119,121] Moreover, the beneficial effects of vitamin D-induced autophagy have been postulated to be exerted via attenuation of inflammation associated with tumorigenesis. However, because autophagy effects can promote both cell survival and inhibition of tumor progression, albeit under different cellular environments, the balance between both these opposing mechanisms on vitamin D treatment needs further study. Such studies will help determine the exact sequence and dose of vitamin D treatment relevant as an adjuvant anticancer drug therapy in combination with tumor treatments [36,122,123,124].

### 2.6. Lessons from Animal Models

Animal models offer additional supportive evidence that demonstrates the effective role of vitamin D in preventing uncontrollable hyperplasia and neoplasia leading to tumor formation [24]. Several kinds of animal models support this notion:

- VDR-knockout mouse models of VDR (VDRKO or VDR^−/−^) *vs.* wild-type counterparts (VDRWT)
- Animals supplemented with vitamin D
- Animal models in which tumorigenesis is chemically induced before animals are supplemented with vitamin D or its analogues
- VDRKO models where tumors are implanted or transfected
- knockout animal or heterozygotes of other genes (e.g., APC^min/+^ or nude mice) subsequently supplemented with vitamin D

Two initial VDRKO models have been established and subsequently reproduced by others to characterize the *in vivo* role of VDR in animal models [125,126]. *In vivo*, the effects of vitamin D on cellular proliferation and terminal differentiation differed between tissues; some tissue types showed hypoplasia and impaired development, whereas other tissues displayed clear hyperplasia. For example, the knockout mice, VDR^−/−^, exhibit growth retardation, impairment of bone formation, osteomalacia, alopecia, impaired folliculogenesis and uterine hypoplasia. Furthermore, transfection of mice with Lewis lung carcinoma cells shows that VDR^−/−^ mice display significantly reduced metastasis of lung cancer cells compared with VDRWT animals [127].

In contrast, VDR^−/−^ mice show hyperproliferation in mammary glands, the parathyroid gland and the descending colon. VDR^−/−^ mice displayed colonic cell division and increased expression of cell proliferation markers, PCNA (proliferating cell nuclear antigen) and cyclin D1. Scientists in the Cross lab later confirmed the VDR^−/−^ model as a suitable model of colorectal hyperproliferation and DNA damage [128,129].

Using the VDR^−/−^ mouse, Zinser and colleagues [130] demonstrated the role of vitamin D and its receptor in mammary gland morphogenesis. Comparing VDR^−/−^ mice with VDRWT, they showed that mammary glands from developing VDR mice at puberty were heavier and exhibited faster growth and ductal branching than their VDRWT counterparts. In organ culture, mammary tissues from VDR^−/−^ treated with estrogen or progesterone exhibited greater hormonal sensitivity than VDRWT. After pregnancy and lactation, VDR^−/−^ mice showed delayed breast gland involution after offspring weaning compared with VDRWT. Mehta and colleagues [131,132] demonstrated in rodent mammary organ culture that treatment with a noncalcemic vitamin D analogue significantly reduces tumor lesion incidence at both the early initiation and promotion stages. In dietary supplementation studies, Jacobson and colleagues [133] demonstrated in dimethylbenzanthracene (DMBA)-treated rats that rats fed on low vitamin D diets had higher tumor incidence than those fed vitamin D-replete diets. VanWeelden and colleagues transplanted nude mice (nu/nu mice) with MCF-7 breast cancer xenografts and, then, followed the treatment with supplementation. Mice were either supplemented with the low-calcemic vitamin D analogue (EB1089) or placebo pellets given to corresponding control subjects. This supplementation lasted for five weeks. In animals administered the vitamin D analogue, cell proliferation in tumors was reduced two-fold compared with control mice. Moreover, the rate of apoptosis was enhanced in EB1089-treated mice, which also experienced significant regression of implanted breast tumors compared with control subjects [134]. These studies indicate that vitamin D action via the VDR is essential to modulate mammary gland proliferation and induce differentiation [130,135,136]. Moreover, such studies indicate an *in vivo* cytoprotective role for vitamin D (and its analogues) in cancer prevention [134,137]. Xu and colleagues suggest through their studies on APC^min/+^ heterozygous mouse models that the *in vivo* growth-modulating effect of vitamin D might be a result of a signaling interplay that allows vitamin D to block the mitogenic effects of growth factors implicated in neoplasty [138,139].

Moreover, deletion of the VDR gene in VDR^−/−^ mice predisposes these mice to higher risk and susceptibility to chemically-induced cancers of the skin, prostate, blood, lymph and breast. For example, treatment of VDRKO mice with DMBA dysregulates skin proliferation and hyperproliferates both basal and epidermal cells with reduced epidermal differentiation. DMBA induced higher incidence of cutaneous lesions in VDR^−/−^ mice than in VDRWT [140,141,142]. However, VDR^−/−^ mice do not spontaneously develop skin tumors without predisposition to carcinogens. Upon DMBA treatment, VDRKO mice also exhibit higher incidence of alveolar and ductal hyperplasias and 16% higher lymphoblastic and thymic lymphomas than VDRWT counterparts. No observed tumor development occurred in tissues, such as ovary, uterus, lung and liver [130,142]. Similarly, in LPB-Tag transgenic mice, pRB and p53 tumor-suppressor genes are inactivated, causing prostate cancer, cellular proliferation and tumor progression much faster in VDRKO mice than in VDRWT. These differences are abolished upon treatment with testosterone. This finding indicated that vitamin D has a growth-modulating effect on cell growth through measured signaling interplay with other growth factors [143].

### 2.7. Evidence for Vitamin D and Cancer

The solar ultraviolet-B (UVB)-vitamin D-cancer hypothesis was proposed by the brothers Cedric and Frank Garland after seeing the atlas of colon cancer mortality rates (MRs) in the United States as beginning graduate students at Johns Hopkins University in 1974. They recognized a general inverse correlation between the regional doses of sunlight and colon cancer MRs. Their seminal paper was published in 1980 [144]. They later added breast [145] and ovarian [146] cancer to the list of cancers with reduced MRs in regions of higher solar radiation.

They used the ecological approach, which compares disease outcomes averaged by geographical units with risk-modifying factors, providing perhaps the strongest evidence that solar UVB and vitamin D reduce the risk of cancer. The evidence has been reviewed in recent papers [147,148]. The reasons the ecological approach is strong for cancer include: that many cases are included, that solar UVB, through production of vitamin D, is a significant risk reduction factor for many types of cancer, that other risk-modifying factors, such as ethnic background and smoking, can be used in the analyses and that cancers develop slowly.

Single-country ecological studies have provided strong and relatively consistent findings for many types of cancer. Single countries have the advantages for ecological studies that the populations are reasonably homogeneous, that they have similar diets and smoking habits, *etc.*, but if not, the differences can be easily modeled, as has been done for the United States in an ecological study that used solar UVB doses for July, lung cancer MRs as the index of smoking and alcohol consumption, those of Hispanic heritage included in the category “white Americans”, poverty level and urban/rural residence [149]. The findings for UVB were similar to those found in an earlier study that used only UVB doses, but omitted several states with high Hispanic population rates [150]. The results for alcohol consumption, Hispanic heritage and smoking were in good agreement with the journal literature. 

There have been several solar UVB indices used in single-country ecological studies: annual solar radiation doses [144,151], solar UVB from the Total Ozone Monitoring System (TOMS) operated by NASA [149,150,152,153,154] and latitude [155,156,157,158,159,160]. A new set of solar UVB dose by month for North America is also available [161]. In some studies, indices of personal UVB irradiance were used: non-melanoma skin cancer in Spain [157] and California [159] and lip cancer less lung cancer in Nordic countries [162]. No factor other than vitamin D production has been proposed to explain the inverse correlations between the solar UVB indices and cancer incidence and/or MRs except for prostate cancer. It was noticed that the geographical variation of prostate cancer MR in the United States shared many features with the atlas of greatest ancestry by county for 2000 [163]. That finding inspired a multi-country ecological study involving genetics, diet and socioeconomic status. The hypothesized genetic link was to the apolipoprotein E epsilon4 (ApoE4) allele, an important risk factor for Alzheimer’s disease [164]. Two important functions of this allele are to increase the production of insulin and cholesterol in order for those with this allele to be able to store more of the excess energy consumed in order to last between feasts. This allele is more common among hunter-gatherer people and those living at high northern latitudes. Several dietary supply factors were considered, and the energy derived from cereals/grains was found to be inversely correlated with prostate cancer incidence and MRs. The index of socioeconomic status used was gross national product per capita. Multiple linear regression analyses using these three factors found an adjusted R of about 0.5, with all three factors having significant correlations with prostate cancer, ApoE4 and gross national product (GNP)/capita as risk factors [164]. Numerous studies have investigated the role of genes in the risk of prostate cancer. Only one other study identified ApoE4 as a risk factor, however [165]. Observational studies generally do not find that serum 25-hydroxyvitmain D (25(OH)D) levels are associated with the risk of prostate cancer [166].

Table 2 summarizes the findings from various single-country ecological studies.

**Table 2:** Cancers for which inverse correlations between incidence and/or mortality rates (MRs) were found with respect to indices of solar UVB dose in single-country studies (references to be supplied).

Cancer	MR (deaths/100,000/Year) *	US [149,152]	Australia [155]	China [153,156]	France [158]	Japan [151]	Nordic countries [162]	Spain [157]
Lung	69.4			X			X	X
Breast	26.9	X	X	X	X		X	X
Colorectal	24.5	X	X	X	X	X	X	X
Prostate	22.0	X	X				X	
Colon	20.1	X	X	X	X	X	X	X
Pancreatic	10.2	X	X			X	X	X
Leukemia	8.8	X	X	X		X		
Ovarian	8.4	X	X					X
Gastric	7.3	X	X	X		X	X	X
non-Hodgkin’s lymphoma (NHL)	7.0	X	X					X
Bladder	6.6	X		X			X	X
Brain	5.2							X
Renal	4.9	X					X	
Esophageal	4.8	X	X	X	X	X	X	X
Rectal	4.4	X		X		X	X	X
Oral, pharyngeal	4.0	X					X	
Endometrial	3.7	X			X			X
Cervical	3.2			X	X			
Gallbladder	1.1	X				X	X	X
Hodgkin’s lymphoma	1.1	X						X
Thyroid	0.4	X						X
Vulvar	0.3	X						

* [167]; MR, MR for males, United States, 1970–94, unless for a female cancer.

The types of cancer are ordered by the MR for males (females for female cancers) for the United States for the period 1970–1994 [167]. The reason for doing so is that the more common a type of cancer is, the easier it is to include enough cases in the study to find significant correlations with solar UVB or vitamin D [168]. As expected, the types of cancer with the higher MRs generally have more findings of inverse correlation with solar UVB doses. For those with fewer significant findings relative to others with similar MRs, this suggests that the beneficial effect of UVB and vitamin D is weak or nonexistent, such as for brain cancer. For those with a relatively higher number of findings for its MR range, it is likely that the effects of UVB and vitamin D are strong, such as for esophageal and gallbladder cancer.

These findings are discussed in greater detail in Grant [147]. Several things can be inferred from the table. One is that the types of cancer with the higher MRs are generally more likely to have been found inversely correlated with the solar UVB indices. This is reasonable, since the uncertainty of each value is reduced [168]. There is little support for vitamin D reducing the risk of brain cancer, as it is a relatively common cancer, but with only one supporting study. On the other hand, the findings for thyroid and vulvar cancers are considered good evidence of the protection by vitamin D, since the MRs are so low.

While ecological studies may provide the earliest and, in some respects, best evidence for a role of solar UVB and vitamin D in reducing the risk of cancer, such findings should be supported by other types of studies in order to evaluate the findings from ecological studies. The next most common type of epidemiological study used to evaluate the role of solar UVB and vitamin D in reducing the risk of cancer is the observational study. There are two types of observational studies: case-control studies in which serum the 25(OH)D level is determined near the time of cancer diagnosis; and nested case-control studies from cohort studies. The perceived advantages of the nested case-control studies include unbiased matching of controls and using 25(OH)D levels, UVB doses or irradiances not affected by the health outcome. Case-control studies could have serum 25(OH)D levels affected by the disease state. However, there does not appear to be any evidence that the existence of cancer per se affects serum 25(OH)D levels or behavior, since most people do not know they have cancer until it is diagnosed. The primary problem of the cohort study approach is that serum 25(OH)D levels from blood drawn at time of enrollment are generally used in the analysis, no matter how many years have elapsed since enrollment. As shown in a pair of papers that examined health outcome with respect to years after enrollment, linear declines were found in risk reduction with respect to follow-up time for cancers [169] and all-cause MR [170].

Table 3 presents an overview of the findings from observational studies supporting the UVB-vitamin D-cancer hypothesis.

**Table 3:** Results from observational studies of cancer incidence with respect to UVB irradiance or serum 25(OH)D levels.

Cancer	[171]	[154]	[172]	Others
Bladder	X *	X	X	[173]
Brain				
Breast				Case-control
Colon		X		Cohort
Colorectal	X			Cohort
Endometrial				[174]
Esophageal	X			
Esophageal, squamous cell		X *		
Gastric	X *		X *	
Head and neck			X	[175]
Hepatoblastoma				[160]
Leukemia	X			
Leukemia, acute lymphoblastic				[160]
Liver			X *	
Lung	X *		X	
Lung, adeno, squamous cell		X		
NHL	X *	X		[160,176]
Oral/pharyngeal	X			
Ovarian				[177]
Pancreatic	X	X	X *	[178]
Pleura		X		
Prostate	X *	X		
Rectal		X *		Cohort
Renal	X *	X	X	[179]
Thyroid		X		[180]

* Not statistically significant.

It is noted that the study from the Health Professionals Follow-up Study [171] and the National Institutes of Health-American Association of Retired Persons (NIH-AARP) Diet and Health study [154] were based, in large part, on solar UVB doses. That was explicit in Lin [154] and implicit in Giovannucci [171] in that it used a modeled serum 25(OH)D level based on measurements of serum 25(OH)D levels in 1000 men with respect to such factors as geographical location, skin pigmentation and leisure time spent out of doors. Other studies based on UVB were those for endometrial cancer [181], hepatoblastoma, acute lymphoblastic leukemia and NHL [160,176].

For colorectal cancer, cohort studies with follow-up times out to 12 years found a significantly reduced risk for higher serum 25(OH)D levels [158]. However, for breast cancer, significantly reduced risk for higher serum 25(OH)D levels was reported only for follow-up times less than three years [169]. The reason for the different findings is that breast cancer is a rapidly developing cancer, while colorectal cancer is not. For example, breast cancer is diagnosed more frequently in spring and fall [182]. The authors suggested that vitamin D reduces breast cancer risk in summer, while melatonin does in winter, due to low sunlight levels.

### 2.8. Clinical Trials

There have been two randomized controlled trials (RCT) using vitamin D and calcium that found a beneficial effect in reducing cancer incidence. The first one was one conducted on post-menopausal women in Nebraska [183]. Those in the treatment arms took 1450 mg/day calcium or 1450 mg/day calcium plus 1100 IU/day vitamin D_3_. At the time of enrollment, the mean serum 25(OH)D level was 72 nmol/L. At the end of the first year, those taking vitamin D plus calcium had a mean serum 25(OH)D level of 96 nmol/L, while those in the other two arms had 71 nmol/L. Between the ends of the first and fourth years, those taking only calcium had a 44% reduction in all-cancer incidence, while those taking calcium plus vitamin D had a 77% reduction. Based on the relationship between breast cancer incidence rates from case-control studies *vs.* serum 25(OH)D level, the expected reduction would be 18%. The difference between 44% and 77% is 33%. Given the uncertainty in both values, and the confounding of vitamin D with calcium, the finding in the RCT is in reasonable agreement with the cancer risk-25(OH)D relation. Calcium has been found to reduce the risk of cancer in a number of studies [184].

The second successful vitamin D/calcium RCT was from a reanalysis of a subset of data from the Women’s Health Initiative study. Participants were given 400 IU/day vitamin D_3_ and 1500 mg/day calcium, but many did not comply and take all of the supplements. For those participants who had not been taking vitamin D or calcium supplements prior to enrolling, there were significant decreases of total, breast and invasive breast cancer of 14%–20% and, nonsignificantly, a reduced risk of colorectal cancer of 17% [185]. For those taking vitamin D and/or calcium prior to enrollment, there was no beneficial effect of taking them during the study. This reduction is about twice that expected from vitamin D alone, so it probably includes a contribution from calcium.

There are a number of reasons why there are so few vitamin D RCTs reporting a reduced risk of cancer. One is that for many years, researchers used 400 IU/day vitamin D_3_. This amount reduces the risk of rickets, but not much else. It is only in the past decade that many of the non-calcemic benefits of vitamin D were reported. For non-calcemic benefits, higher serum 25(OH)D levels are required. A second reason is that most of the vitamin D RCTs treated vitamin D as a drug, assuming that other sources of vitamin D were unimportant and that there was a simple dose-response relation between oral vitamin D and the serum 25(OH)D level. In fact, the vitamin D-serum 25(OH)D level relation is nonlinear and eventually saturates [186]. Proper vitamin D RCTs should have a model relationship between serum 25(OH)D level and health outcome, generally from observational studies, enroll people at the lower end of this relationship, supplement them with sufficient vitamin D_3_ to raise their serum 25(OH)D level to the upper end and measure serum 25(OH)D levels at the time of enrollment and after a year of supplementing, as well as consider other sources of vitamin D, such as solar UVB irradiance. The guidelines for conducting vitamin D RCTs were outlined in a recent paper [187].

### 2.9. Cancer Survival

Another way to examine whether vitamin D reduces the risk of cancer is to investigate whether those with higher serum 25(OH)D levels at the time of cancer diagnosis have better survival rates than those with lower levels. Those diagnosed with colorectal cancer with serum 25(OH)D levels in the upper quartile had half the MR of those in the lower quartile [188]. Such findings led to a study on the disparities in cancer survival rates for black Americans compared to white Americans [189]. It was found that there are disparities for 13 types of cancer after consideration of socioeconomic status, stage at diagnosis and treatment: bladder, breast, colon, endometrial, lung, ovarian, pancreatic, prostate, rectal, testicular and vaginal cancer, Hodgkin lymphoma and melanoma. Solar UVB doses and/or serum 25(OH)D levels have been reported to be inversely correlated with incidence and/or MRs for all of these cancers. The disparities not accounted for by socioeconomic status, stage at diagnosis and treatment ranged from 0% to 50%, with a mean value near 25%. This value is consistent with the disparities in serum 25(OH)D levels for black and white Americans: 40 nmol/L for black Americans and 65 nmol/L for white Americans [190].

### 2.10. Hill’s Criteria for Causality

A. Bradford Hill laid down the criteria for causality in a biological system in 1965 [191]. The primary criteria appropriate for UVB irradiance and vitamin D include strength of association, consistent findings in different populations, temporality, biological gradient, plausibility (e.g., mechanisms), experiment (e.g., RCT) and analogy. As seen from the information presented in this work, most of the criteria are satisfied to a reasonable extent, especially for breast and colorectal cancer. By invoking analogy, many of the other cancers also qualify based on the findings in ecological studies. Hill’s criteria for causality have been applied to many types of cancer, finding them largely satisfied for breast and colorectal cancer and somewhat satisfied for several other types of cancer [192], as well as for breast cancer [193].

## 3. Recommendations/Conclusions

Based on the evidence reviewed in this paper, there is very good-to-excellent scientific evidence that solar UVB irradiance and vitamin D reduce the risk of many types of cancer. However, acceptance by public policy review boards has not yet accepted the evidence. Part of the reason is that they generally require well-conducted randomized controlled trials rather than ecological or observational studies before making recommendations. Another reason is that UV irradiance is considered the most important risk factor for melanoma and non-melanoma skin cancer, so many public health bodies are reluctant to recommend more sun exposure.

## Abbreviations

WSTF: Williams syndrome transcription factor
WINAC: WSTF including nucleosome assembly complex
MCF-7: Michigan Cancer Foundation-7 human breast adenocarcinoma cell line
MART-10: 19-nor-2α-(3-hydroxypropyl)-1α,25-Dihydroxyvitamin D3
EB1089: Seocalcitol
SW620: Human colorectal adenocarcinoma cell line
PC/JW: Human colorectal adenoma-derived epithelial cell line derived from adenomatous polyposis
HT29: Human colorectal adenocarcinoma cell line HT29
SW-480-ADH: malignant colon cancer subline of human Dukes’ type B colorectal adenocarcinoma cell line SW-480
LNCaP: Human prostatic carcinoma cell line LNCaP
CL-1: Human prostate cancer cell line derived from LNCaP
IGFBP3: IGF-binding protein 3
MCF10CA: Human metastatic breast cancer cell line MCF10CA
HL60: Human promyelocytic leukemia cells
mTOR: mammalian target of rapamycin
CDK: Cyclin-dependent kinase
APC: Adenomatous polyposis coli
LPB-Tag: Long Probasin Promoter-Large T Antigen
